# Do we need new trials of procalcitonin-guided antibiotic therapy? A response

**DOI:** 10.1186/s13054-018-2008-y

**Published:** 2018-03-23

**Authors:** Jos A. H. van Oers, Maarten W. Nijsten, Dylan W. de Lange

**Affiliations:** 1 Department of Intensive Care Medicine, Elisabeth Tweesteden Ziekenhuis, P.O. Box 90151, 5000 LC Tilburg, the Netherlands; 2 Department of Critical Care, University Medical Center Groningen, University of Groningen, Groningen, the Netherlands; 3 Department of Intensive Care Medicine, University Medical Centre Utrecht, Utrecht, The Netherlands

Antibiotic treatment needs to be short, appropriate (focused on the right pathogen), and adequate (at the right dosage). And still, many physicians treat patients for too long. A recent meta-analysis on procalcitonin (PCT)-guided antibiotic treatment in acute respiratory infections [1] showed that antibiotics could be shortened form 8.1 to 5.7 days. The key question is, why do physicians treat for so long? The answer may be fear! Fear of undertreatment.

We read with great interest the commentary by Lisboa and colleagues in *Critical Care* [2] in which they question the clinical utility of this meta-analysis [1]. They concluded that populations in previous trials were not receiving best care, had less adherence to PCT algorithms, and lacked information on specific conditions and populations. As authors of the largest study included in this meta-analysis, the Stop Antibiotics on Procalcitonin guidance Study (SAPS) [3], we want to respond. SAPS was a pragmatic randomized controlled trial in the Netherlands with 1546 adult ICU patients with antibiotics for a presumed infection. We demonstrated a highly significant reduction in initial antibiotic duration (5.0 vs 7.0 days). The median duration of antibiotic treatment (DOT) in the control group of the total population was 7 days (interquartile range (IQR) 4–11 days). Of these patients, 65% had a presumed pulmonary infection. Dutch national guidelines recommend an antibiotic duration for moderate-severe community-acquired pneumonia (CAP) of 5 days [4]. No such advice exists for severe pneumonia admitted to the ICU. The median DOT in the control group in CAP was 7 days (IQR 4–10 days), 6 days (IQR 4–10 days) in hospital-acquired pneumonia and 7 days (IQR 5–11 days) in ventilator-associated pneumonia. The wide IQR suggests that physicians are reluctant to trust guidelines and prefer to prolong antibiotic treatment if they believe it is necessary. Moreover, physicians may perform even better in clinical trials, because they know they are being watched, commonly referred to as the “Hawthorne effect”. In SAPS the patients were already on antibiotics. When a PCT-stopping criterion was reached antibiotics were stopped in 53% of the patients within 48 h. It was a stopping advice. Sensitivity and specificity are not high enough to withhold antibiotics on PCT alone. And indeed, PCT is no holy grail. Like other biomarkers, there are numerous non-infectious inflammatory processes, i.e., trauma, surgery, and acute kidney injury, in which PCT can be elevated. But such conditions were well balanced between both groups.
